# Evaluation of Chagas heart disease by cardiac magnetic resonance after an aborted sudden cardiac death event

**DOI:** 10.1186/1532-429X-14-S1-P176

**Published:** 2012-02-01

**Authors:** Gustavo J Volpe, Henrique S Trad, Marcel Koenigkam-Santos, Henrique T Moreira, Benedito C Maciel, Jose A Marin-Neto, Andre Schmidt

**Affiliations:** 1 Internal Medicine, Medical School of Ribeirao Preto-University of Sao Paulo, Ribeirao Preto, Brazil; 2 Internal Medicine - Radiology Division, Medical School of Ribeirao Preto-University of Sao Paulo, Ribeirao Preto, Brazil

## Background

Chagas’ disease (CD) is caused by the protozoan parasite Trypanosoma cruzi leading to a lifelong infection, which is still recognized as one of the world’s most neglected tropical diseases. After an acute phase, which is commonly unapparent, 30-40% of the patients develop the chronic form and chronic Chagas 'cardiomyopathy (CCC), its most serious complication, has three main clinical presentations: arrhythmic syndrome, heart failure, and thromboembolic phenomena. All of these forms can lead to the most important outcome in CCC: the sudden cardiac death (SCD). This dramatic event can affect even young and previously asymptomatic patients. Cardiac Magnetic Resonance (CMR) is becoming a valuable tool to evaluate and stratify patients regarding the risk of severe arrhythmic events in many clinical conditions. Little is known about its prognostic value in CCC. Our objective was to report a series of aborted SCD in CCC patients in order to clarify if CMR can detect a common imaging pattern in such cases.

## Methods

We evaluated five chagasic patients (all male, 49,2 ± 10,2 years old) referred to our institution after an aborted SCD episode between September/2009 and November/2010, before an implantable cardiodefibrillator (ICD) procedure. All patients underwent coronary angiography to exclude coronary artery disease. All of them underwent a CMR exam with cines and delayed enhancement sequences with gadolinium before the insertion of ICD and a clinical follow-up until September 2011 was completed for all patients.

## Results

All patients were asymptomatic before and after the event (NYHA class I). LVEF was depressed in only two patients (both with 32%) and all had mildly depressed RVEF (52,4 ± 9,45%). In three patients, including the two with depressed left ventricular function, LVED diameter was increased. None of them had an apical aneurysm (a characteristic finding in CCC) but all presented myocardial hyperenhancement areas in delayed enhancement sequence. The number of segments involved varied from 2 to 6 in each patient, three of them showed transmural pattern and the posterior-lateral wall (3 patients) was the preferential location. After a mean follow-up of 392 days, all patients received at least one appropriate ICD therapy. At the last follow-up consultation, all patients were taking amiodarone and maintained NYHA class I functional status.

## Conclusions

LVEF or NYHA status do not appear to be good prognostic markers of SCD in CCC, but the presence of extensive myocardial fibrosis, as shown in a CMR, exam may be a necessary substrate for that event.

## Funding

None.

